# Case report: An intriguing case of Philadelphia chromosome–positive acute lymphoblastic leukemia recurrence

**DOI:** 10.3389/fonc.2024.1457832

**Published:** 2024-07-31

**Authors:** Nicolò Peccatori, Elena Chiocca, Valentino Conter, Annalisa Tondo, Matilde Marzorati, Tommaso Casini, Marinella Veltroni, Andrea Biondi, Grazia Fazio

**Affiliations:** ^1^ Tettamanti Center, Fondazione IRCCS San Gerardo dei Tintori, Monza, Italy; ^2^ Pediatrics, Fondazione IRCCS San Gerardo dei Tintori, Monza, Italy; ^3^ School of Medicine and Surgery, University of Milano-Bicocca, Monza, Italy; ^4^ Division of Pediatric Oncology/Hematology, Children’s Hospital A. Meyer IRCCS, Florence, Italy

**Keywords:** acute lymphoblastic leukemia, Philadelphia (Ph)/BCR-ABL positive, e1a3, *BCR::ABL1*, extramedullary relapse, second malignant neoplasm (SMN), T-cell lymphoblastic leukemia or lymphoma, Ig/TCR gene rearrangement

## Abstract

The incorporation of tyrosine kinase inhibitors (TKIs) in the treatment of Philadelphia chromosome–positive acute lymphoblastic leukemia (Ph+ ALL) led to significant improvement. However, in the pediatric setting, the outcomes of Ph+ ALL are still inferior compared to those of other ALL subtypes even in the TKI era due to higher relapse rate. Herein, we report a very peculiar case of late extramedullary Ph+ ALL relapse in a child, characterized by lymphomatous presentation in the tonsils and lymphoid lineage switch. The diagnostic dilemma between the occurrence of a second malignant neoplasm and the recurrence of the primary disease is further discussed, highlighting the importance of molecular backtracking analysis. This case report emphasizes the high plasticity and polyclonal nature of ALL and expands the heterogeneity of possible clinical presentation of Ph+ ALL at relapse.

## Introduction

Philadelphia chromosome–positive acute lymphoblastic leukemia (Ph+ ALL) is defined by the translocation t(9;22)(q34; q11,2) and accounts for 2%–5% of pediatric and 25%–30% of adult ALL cases (1). Of note, Ph+ ALL is almost exclusively of B lineage, with less than 2% of cases of T lineage in the pediatric Children Oncology Group (COG) and in the Ph+ ALL European group (EsPhALL) studies (2–5). The incorporation of molecularly targeted therapy with ABL-directed tyrosine kinase inhibitors (TKIs) in its treatment has led to dramatic survival improvements (6). Nevertheless, in the pediatric setting, the outcomes of Ph+ ALL are still inferior compared to those of other ALL subtypes even in the TKI era, due to higher cumulative incidence of relapse (2–5). The reported overall relapse rates in the major international pediatric Ph+ ALL trials incorporating TKIs were 28%, 26%, 25%, and 35% for EsPhALL2004 (2), EsPhALL2010 (3), AALL0622 (4), and CA180–372/COG AALL1122 trial (5), respectively. Among relapsed patients, the rate of extramedullary relapse excluding central nervous system (CNS) relapse was overall rare with three cases (6%) of isolated/combined testis relapse in EsPhALL2004, one case (2.5%) of combined bone marrow (BM) plus eye relapse in EsPhALL2010, one case (4%) of isolated axillary relapse in AALL0622, and six cases (15%) of isolated/combined extramedullary relapses in CA180–372/COG AALL1122. Herein, we present a case of extramedullary relapse of Ph+ ALL in an unusual site and with a lymphoid lineage shift.

## Case description

A 7-year-old child presented with a 3-week history of fatigue, arthralgias, and hepatosplenomegaly. The complete blood count (CBC) showed a white blood cell count of 8.5 × 10^9^/L with a predominance of atypical lymphocytes, hemoglobin 10 g/dL and a platelet count of 133 × 10^9^/L. The BM aspirate revealed a massive infiltration of abnormal lymphoblasts (66%) expressing CD19, CD10, CD34, and CD20 and negative for CD3, CD5, and CD7, consistent with a diagnosis of B-cell precursor acute lymphoblastic leukemia (BCP-ALL). No leukemic blasts were found in the cerebrospinal fluid (CSF), and no other extramedullary localization of the disease was detected. Cytogenetics showed the presence of the translocation t (9,22)(q34; q11.2), and molecular testing was positive for the *BCR::ABL1* p190 transcript (e1a3 variant), establishing the diagnosis of Ph+ BCP-ALL. A four-drug induction with prednisone, vincristine, daunorubicin, and Pegylated Asparaginase (PEG)-asparaginase was initially started according to the AIEOP-BFM ALL 2017 protocol (EudraCT 2016–001935–12 and NCT03643276). On day +15, imatinib was added, and, on day +22, the patient was enrolled in the EsPhALL2017/COG AALL1631 study (EudraCT 2017–000705–20 and NCT03007147). End-of-consolidation (EOC) minimal residual disease (MRD), assessed by immunoglobulin T-cell-receptor PCR (IgH/TCR-PCR), was undetectable. Treatment was discontinued 2 years after the diagnosis.

**Figure 1:**
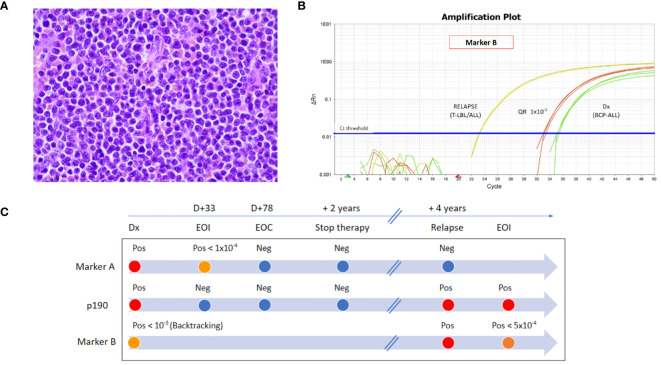
**(A)** Histology (H&E) of the tonsil at disease recurrence. **(B)** RT-qPCR amplification plot of marker TCR Dd2.Dd3 (Marker B) at first diagnosis by backtracking analysis in BM (green curves) and at disease recurrence in the tonsil (yellow curves). Quantitative range 1 × 10^−3^ (red curves). **(C)** Timeline of the disease monitoring by RT-qPCR with marker IgH.DH3.JH6 (Marker A), by RT-PCR of p190 *BCR::ABL1* transcript (P190), and by RT-qPCR with marker TCR Dd2.Dd3 (Marker B). Abbreviations: Dx, diagnosis; D, day; EOI, end-of-induction; EOC, end-of-consolidation; Pos, positive; Neg, negative; T-LBL, T-cell lymphoblastic lymphoma; BCP-ALL, B-cell precursor acute lymphoblastic leukemia.

Four years after the diagnosis, the patient presented with fever, cervical lymph adenopathy, and bilateral severe exudative tonsillar swelling. Diagnostic work-up showed high Epstein–Barr virus (EBV) copy number in peripheral blood (PB) (1.926.350 copies/mL), consistent with EBV infection. Chest X-ray and abdominal ultrasound were normal, and CBC did not show any abnormality. Broad-spectrum antibiotic therapy was started, and prednisone was added because of the risk of upper airway obstruction. Clinical course was characterized by progressive exudative tonsillar hypertrophy causing obstructive sleep apnea syndrome and by enlargement of the lateral cervical and inguinal lymph nodes. Adeno-tonsillectomy was thus performed, and histological analysis of the tonsils revealed a diffuse lymphoid infiltrate with small- to medium-size blast cells with mitotically active features, finely dispersed chromatin, and scant cytoplasm (**Figure 1A**). Immunohistochemical findings showed positivity for TdT, CD3 CD5, CD7, CD4, CD79a, CD38, CD19 (weak), and CD10 (weak) and negativity for CD20 and MPO. Proliferative index (Ki67) was 50%. Morphological and immunohistochemical features were consistent with the diagnosis of T-cell lymphoblastic lymphoma (T-LBL). The PET-scan confirmed the presence of high metabolic activity in the oropharynx and in cervical and inguinal lymph nodes. BM aspirate was thus performed and showed 3% of T-lineage lymphoblasts (cyCD3+, CD7+, CD5+, CD45+, CD38+, CD4−, CD8−, CD56−, and CD34−). No blasts were found in PB at morphological assessment or in CSF by microscopic examination of cytospin. RT-PCR detected p190 *BCR::ABL1* (e1a3) positivity, both in PB and BM. Hence, RNA was extracted from the biopsy tonsil sample, and RT-PCR for *BCR::ABL1* was performed, confirming the positivity for the same p190 e1a3 *BCR::ABL1* transcript. RT-PCR for *BCR::ABL1* transcript was also performed on BM samples of end-of-induction, EOC, and treatment discontinuation time points at first diagnosis and resulted negative.

At initial diagnosis, two IgH rearrangements (IgH.DH3.JH6 and IgH.VH3.JH4) were identified by next-generation sequencing (NGS) marker screening strategy (7), and MRD monitoring by real-time quantitative polymerase chain reaction (RT-qPCR) showed negativity for both MRD markers during maintenance and at the time of treatment discontinuation. To ascertain if the patient presented with a recurrence of the primary disease, RT-qPCR for these two rearrangements was performed on tonsils and BM samples, and both resulted negative. However, T lymphoblasts detected in the tonsil presented a different unique rearrangement by NGS (i.e., TCR Dd2.Dd3), which was identified also in 2.5% of cells in the contemporary BM. This latter marker was not identified at the initial diagnosis by marker screening (reads cutoff >5%). To verify if a blast population positive for this marker was already present at initial diagnosis, backtracking RT-qPCR analysis was performed and showed a low-level positivity (not quantifiable, QR 10–3) (**Figures 1B, C**).

Treatment of the patient after disease recurrence consisted of high-dose cytarabine, fludarabine, and doxorubicin (FLA) associated with dasatinib. CNS-directed prophylaxis with triple intrathecal chemotherapy including methotrexate, cytarabine, and methylprednisolone was administered. End of re-induction MRD resulted positive-non-quantifiable (<5 × 10^−4^, marker TCR Dd2.Dd3), and the PET-scan showed complete metabolic response. The patient shall proceed to allogeneic stem cell transplantation shortly.

## Discussion

In this report, we describe a pediatric patient firstly diagnosed with Ph+ BCP-ALL and treated with a TKI-containing multiagent chemotherapy regimen who developed a late T-lineage extramedullary relapse characterized by lymphomatous presentation in the tonsils, and, to the best of our knowledge, this is the first such case to be reported. Interestingly, the disease recurrence was associated with confounding features, i.e., the concomitant EBV infection, which *per se* could explain the clinical presentation.

The unusual clinical presentation and the molecular features of this case posed a diagnostic dilemma over the pathogenesis of the disease recurrence. The switch from B- to T-lineage ALL and the absence at disease recurrence of the IgH rearrangements detected at initial diagnosis might suggest the occurrence of a second malignant neoplasm (SMN). On the other hand, the persistence of the Ph+ chromosome and, especially, the identical *BCR::ABL1* rearrangement (p190 e1a3) were consistent with the recurrence of the primary disease. Of note, e1a3 *BCR::ABL1* is an extremely rare transcript variant, reported only anecdotally in ALL cases. It is a result of the translocation between exon 1 of *BCR* on chromosome 22 and exon 3 of *ABL1* on chromosome 9, and its clinical significance is still unclear (8). As a general consideration, before diagnosing a secondary hematological malignancy, it is crucial to rule out the emergence of minor subclones not identified at the diagnosis of the primary disease. Of note, in our case, backtracking molecular analysis by RT-qPCR provided very important insights. The unique TCR rearrangement that characterized the T-LBL was already present at the initial diagnosis of BCP-ALL, although, in a minor subclone, likely representing less than 0.1% of the blast population, indicating a clonal relation between the two entities. We do not have, however, the possibility to establish if this minor subclone was of T lineage or not. Notably, this relevant molecular information would not have been accessible without the availability of stored biological material from the patient, underpinning the crucial role of biobanking in the optimal diagnostic and clinical management of hematological malignant diseases.

Flow cytometric studies of the T-LBL blasts showed an aberrant weak expression of multiple B-lineage markers such as CD79a, CD10, and CD19, suggesting the occurrence of a lineage switch. Lineage switch describes the condition where acute leukemia converts during therapy or at relapse to a different lineage (B-/T-lymphoid lineage or myeloid lineage) compared to that expressed at diagnosis (9). It generally occurs in the context of ambiguous lineage acute leukemias, comprising mixed-phenotype acute leukemia and bilineal acute leukemia, and is typically associated with specific genetic subtypes of ALL, such as *KMT2A*r ALL (10). Most cases of lineage transformation involve a switch from ALL to acute myeloid leukemia, but it can be bidirectional and albeit extremely rarely from B to T lineage and vice versa (11). The mechanisms involved in lineage switch in acute leukemia are still unclear, and different hypotheses have been proposed to explain this phenomenon, including clonal evolution of multipotent or multilineage original blastic progenitor and therapy-mediated selection of lineage-committed subclones. Of interest, modern B-cell targeting treatment strategies, such as anti-CD19–bispecific T-cell engager Blinatumomab or CD19 CART cell therapies, may further emphasize this phenomenon (12–14).

Furthermore, despite of the rarity of extramedullary relapse in patients with Ph+ ALL, the event of SMN in this context is even more rare. The incidence of SMN after treatment of childhood ALL was reported to be around 1% of cases, and only a minority of them developed hematologic SMN (15). Overall, in the published pediatric Ph+ ALL trials, SMN was reported as an event only in two cases (4, 5).

In conclusion, the cytogenetic, flow-cytometric, and molecular analysis in our case indicated a common clonal origin between the recurrence of T-ALL/LBL and the initial BCP-ALL diagnosis. Although we do not have the conclusive proof dissecting the pathogenesis of the disease recurrence, i.e., the genomic concordance of *BCR::ABL1* breakpoint sequencing between diagnosis and relapse, the findings in our case are consistent with a leukemogenesis process involving the selection of a common malignant progenitor cell capable of pluripotent differentiation. This case report emphasizes the high plasticity and polyclonal nature of ALL and expands the heterogeneity of possible clinical presentation of Ph+ ALL at relapse. Notably, it shows that backtracking analysis may allow to discern between the occurrence of a SMN and the recurrence of the primary disease.

## Data availability statement

The raw data supporting the conclusions of this article will be made available by the authors, without undue reservation.

## Ethics statement

Ethical approval was not required for the study involving human samples in accordance with the local legislation and institutional requirements because [reason ethics approval was not required]. Written informed consent for participation in this study was provided by the participants’ legal guardians/next of kin. Written informed consent was obtained from the individual(s), and minor(s)’ legal guardian/next of kin, for the publication of any potentially identifiable images or data included in this article.

## Author contributions

NP: Writing – review & editing, Writing – original draft, Conceptualization. EC: Writing – review & editing, Conceptualization. VC: Writing – review & editing, Conceptualization. AT: Writing – review & editing. MM: Investigation, Writing – review & editing. TC: Writing – review & editing. MV: Writing – review & editing. AB: Writing – review & editing, Supervision. GF: Writing – review & editing, Conceptualization.
